# Functional comparison of phosphomimetic S15D and T160D mutants of myosin regulatory light chain exchanged in cardiac muscle preparations of HCM and WT mice

**DOI:** 10.3389/fcvm.2022.988066

**Published:** 2022-09-20

**Authors:** Katarzyna Kazmierczak, Jingsheng Liang, Michelle Gomez-Guevara, Danuta Szczesna-Cordary

**Affiliations:** Department of Molecular and Cellular Pharmacology, University of Miami Miller School of Medicine, Miami, FL, United States

**Keywords:** myosin RLC, phosphorylation, phosphomimetic S15D and T160D RLCs, reconstituted cardiac muscle preparations, super-relaxed state of myosin, transgenic mice

## Abstract

In this study, we investigated the rescue potential of two phosphomimetic mutants of the myosin regulatory light chain (RLC, *MYL2* gene), S15D, and T160D RLCs. S15D-RLC mimics phosphorylation of the established serine-15 site of the human cardiac RLC. T160D-RLC mimics the phosphorylation of threonine-160, identified by computational analysis as a high-score phosphorylation site of myosin RLC. Cardiac myosin and left ventricular papillary muscle (LVPM) fibers were isolated from a previously generated model of hypertrophic cardiomyopathy (HCM), Tg-R58Q, and Tg-wild-type (WT) mice. Muscle specimens were first depleted of endogenous RLC and then reconstituted with recombinant human cardiac S15D and T160D phosphomimetic RLCs. Preparations reconstituted with recombinant human cardiac WT-RLC and R58Q-RLC served as controls. Mouse myosins were then tested for the actin-activated myosin ATPase activity and LVPM fibers for the steady-state force development and Ca^2+^-sensitivity of force. The data showed that S15D-RLC significantly increased myosin ATPase activity compared with T160D-RLC or WT-RLC reconstituted preparations. The two S15D and T160D phosphomimetic RLCs were able to rescue V_max_ of Tg-R58Q myosin reconstituted with recombinant R58Q-RLC, but the effect of S15D-RLC was more pronounced than T160D-RLC. Low tension observed for R58Q-RLC reconstituted LVPM from Tg-R58Q mice was equally rescued by both phosphomimetic RLCs. In the HCM Tg-R58Q myocardium, the S15D-RLC caused a shift from the super-relaxed (SRX) state to the disordered relaxed (DRX) state, and the number of heads readily available to interact with actin and produce force was increased. At the same time, T160D-RLC stabilized the SRX state at a level similar to R58Q-RLC reconstituted fibers. We report here on the functional superiority of the established S15 phospho-site of the human cardiac RLC vs. C-terminus T160-RLC, with S15D-RLC showing therapeutic potential in mitigating a non-canonical HCM behavior underlined by hypocontractile behavior of Tg-R58Q myocardium.

## Introduction

Phosphorylation of cardiac sarcomeric proteins is a critical regulator of cardiac muscle contraction and a modulator of the physiological performance of the heart. Among essential phosphorylatable proteins is the regulatory light chain (RLC) of cardiac myosin (*MYL2* gene), which is attached to the myosin heavy chain (MHC) at the distal part of the neck region (lever arm) of the myosin head (1). The RLC, together with the adjacent myosin essential light chain (ELC), provides structural stability to the lever arm and supports an ATP-dependent rotational movement of this region of the myosin head to execute the power stroke and sarcomere shortening (2–4). The N-terminus of myosin RLC comprises a Ca^2+^/Mg^2+^ binding site and myosin light chain kinase (MLCK)-dependent phosphorylation site, both regions capable of altering the alpha-helical structure of the RLC and its Ca^2+^ binding properties (5, 6). Under physiological conditions, the cardiac regulatory light chain is phosphorylated at ~0.4 moles of phosphate per mole of RLC in various species, including humans (7, 8). RLC phosphorylation has been proposed to result in the movement of myosin heads toward thin filaments facilitating cross-bridge formation, accelerating rates of actin-myosin interaction, and increasing the Ca^2+^ sensitivity of force development (9–12). Studies from the Irving group suggest that RLC phosphorylation induces changes in the Ca^2+^ sensitivity of force through structural changes in thin filaments rather than by phosphorylation-induced availability of myosin heads for thin-filament binding (13).

In rodent hearts, RLC can be unphosphorylated or occur in a single or double phosphorylated form at two serine residues, S14 and S15. At the same time, the human ventricular RLC can only be singly-phosphorylated at S15 (14). Using an *in vitro* phosphorylation assay with cardiac MLCK, it was shown that S15 of the human cardiac RLC is the only N-terminal RLC site that is phosphorylated by cardiac MLCK (10). This result confirmed the physiological relevance of the S15-RLC site in the heart.

**Figure 1:**
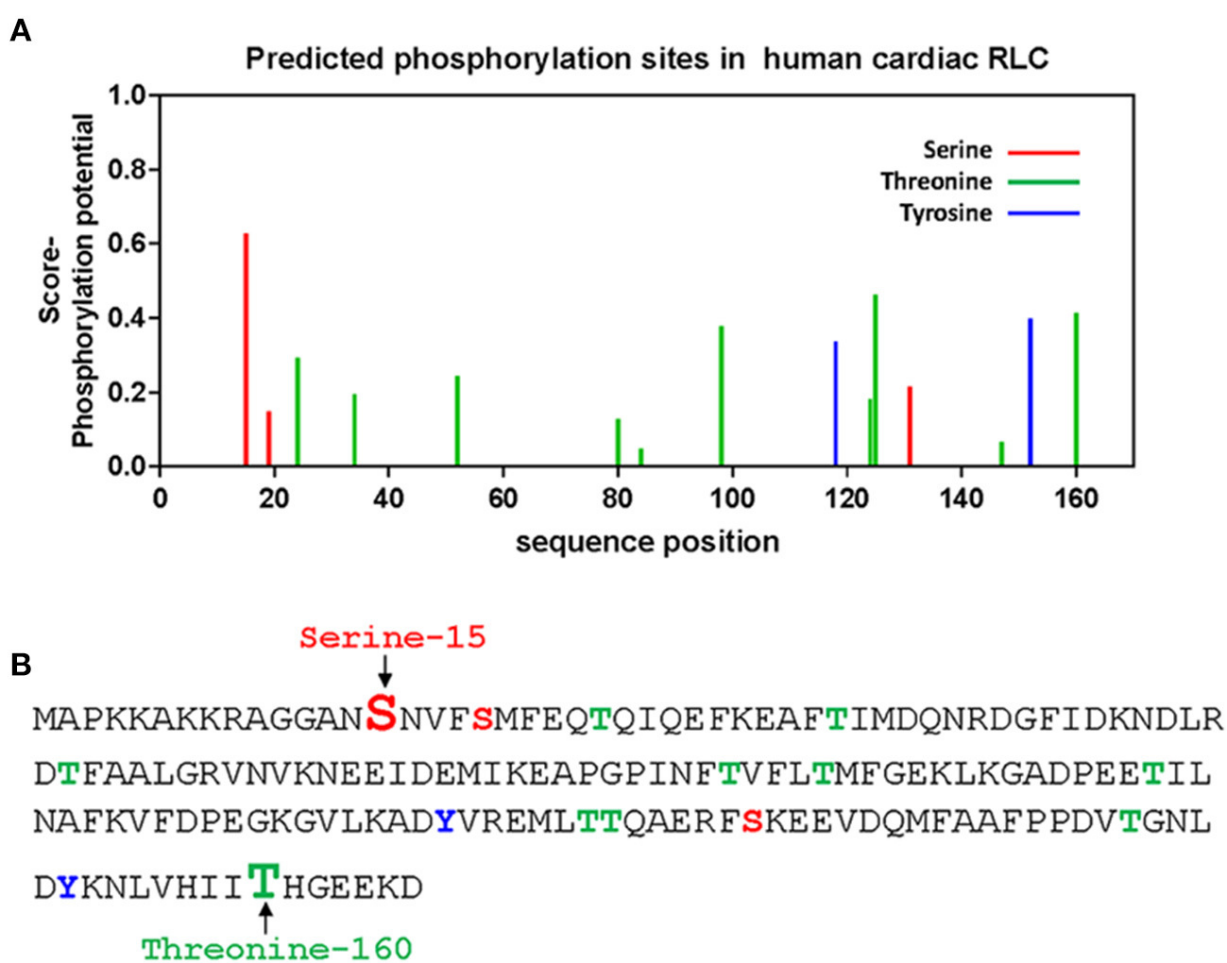
*In-silico* prediction of phosphorylation sites of the human cardiac RLC. **(A)** Phosphorylation of Ser/Tyr/Thr sites in the RLC was predicted by PhosphoSVM search http://sysbio.unl.edu/PhosphoSVM/prediction.php/. **(B)** Amino acid sequence of the human cardiac RLC (NCBI_P10916) with the location of predicted pSer, pTyr, and pThr residues. Bolded and enlarged, the S15 (in red font) and T160 (in green font) sites are studied in this report.

The question that we asked in this investigation was whether there are other phosphorylatable residues in the human cardiac RLC that could play functional roles in actomyosin interaction and cardiac muscle contraction. Besides the established S15 site, *in-silico* analysis identified two new sites at T125 and T160 as highly scored phosphorylatable residues in the human cardiac RLC (15, 16) (Figure 1). Relevant to this investigation is the fact that T160 is localized in the C-terminus of the RLC molecule. This region encompasses many *MYL2* variants associated with hypertrophic cardiomyopathy (HCM). One of the first *MYL2* mutations identified to cause HCM and located in the C-terminus RLC was D166V, where the last amino acid, aspartate-166, was replaced by valine (17). Notably, the D166V mutation was also associated with malignant HCM outcomes. The same aspartate-166 residue was found to be mutated to alanine (D166A) in a cohort of 124 consecutive HCM patients in the study by Alvarez-Acosta et al. (18). The authors also reported on I158L-RLC mutation causing obstructive hypertrophy and atrial fibrillation but with a good prognosis (18). In 2020, D166 residue was again found to be mutated to histidine (D166H) (19). D166H was found among multigenerational family members and appeared to be highly penetrant. A high restrictive filling pattern and atrial fibrillation incidence were observed (19). In the same year, the Garg group reported on another missense *MYL2* variant (G162R) located in the vicinity of T160 in the C-terminus of RLC (20).

Therefore, this investigation focused on the C-terminal T160-RLC residue that comprises several *MYL2* missense mutations of benign to malignant HCM phenotypes (17–20). The goal was to study whether the phosphorylation of T160 *via* the RLC phosphomimetic approach, replacing aspartate for threonine (T160D), would be able to mitigate any of the HCM adverse phenotypes in a mouse model of HCM reconstituted with recombinant human cardiac T160D-RLC. The function of T160D-RLC phosphomimetic was compared to that of S15D-RLC, which was assessed along with T160D and served as a control. We also compared the data on S15D-RLC phosphomimetic to previous studies of S15D-RLC phosphomimetic-induced effects on heart function *in vitro* (21, 22) and *in vivo* (23, 24). Specifically, we previously showed that S15D phosphomimetic in the background of HCM-D166V RLC mutation was able to rescue the binding of D166V-myosin to actin and increased force generation capacity in the *in vitro* motility assays compared with D166V reconstituted porcine myosin (21). Likewise, S15D phosphomimetic in the background of human cardiac HCM-R58Q RLC mutation rescued R58Q-exerted low isometric force and Vmax of actin-activated myosin ATPase activity in S15D-R58Q- vs. R58Q-reconstituted porcine cardiac muscle preparations (22). We also showed improved heart function *in vivo* in HCM-D166V mice injected with AAV9-S15D-RLC (24) and in double transgenic mice expressing the S15D phosphomimetic in the background of HCM-D166V mutation (23).

We cloned and expressed human cardiac T160D and S15D phosphomimetic RLC mutants and tested them in chemically skinned, and RLC-depleted left ventricular papillary muscles (LVPM) and cardiac myosin from transgenic wild-type (Tg-WT) and HCM Tg-R58Q mice. Both mouse models expressed the human cardiac RLC-WT or RLC-R58Q mutant.

The R58Q model displays a non-canonical HCM phenotype that is hypo- rather than hypercontractile and stabilizes the OFF state of myosin in LVPM fibers from Tg-R58Q mice, and in R58Q recombinant protein-reconstituted porcine fibers (22). A similar observation of the R58Q-mediated hypocontractile activity was reported by Kampourakis et al. who demonstrated that R58Q promotes an OFF state that reduces the number of myosin cross-bridges readily available for actin interaction and ATP utilization (25). Using a loaded *in vitro* motility assay, we previously showed an R58Q-mediated decrease in actin sliding velocity resulting in a significant reduction in force production (26, 27). Interestingly, actin sliding velocity and force were restored upon MLCK-induced phosphorylation of the R58Q mutant (26).

In this report, we showed that in Tg-R58Q preparations, both T160D and S15D phosphomimetics -could restore myosin ATPase activity and maximal isometric tension to the level observed for WT-RLC-reconstituted HCM Tg-R58Q preparations. However, S15D-RLC was more efficient than T160D-RLC in activating myosin ATPase activity (V_max_) in Tg-R58Q and Tg-WT hearts. In addition, in LVPM fibers of HCM-R58Q myocardium, S15D-RLC was observed to cause a shift in the myosin energetic states from the super-relaxed (SRX) to disordered relaxed (DRX) state, while T160D-RLC maintained the SRX-to-DRX ratio at the level observed for R58Q-reconstituted fibers. Alterations of cardiac SRX may result in changes in sarcomere force production and energy utilization in the heart (28). Therefore, the S15D-RLC-mediated increase in the DRX heads readily available to interact with actin and produce force suggests that S15D-RLC phosphomimetic may serve as a therapeutic modality to counteract the hypocontractile activity of HCM-R58Q myocardium.

## Materials and methods

### Transgenic mice

This work was performed in accordance with the Guide for the Care and Use of Laboratory Animals published by the U.S. National Institutes of Health (NIH Publication no. 85–23, revised 2011). Animal protocols were endorsed by the Institutional Animal Care and Use Committee at the University of Miami Miller School of Medicine (protocol #21-106 LF). The assurance number is #A-3224-01, approved through November 30, 2023. Mice were euthanized by CO_2_ inhalation that was followed by cervical dislocation.

The characterization of transgenic (Tg) mice, including the determination of Tg protein expression, has been previously described (29). For this study, we used Tg-WT line 2 (L2), expressing ~90% of human cardiac WT-RLC (NCBI_P10916), and two Tg-R58Q lines, L8 and L9, showing ~95% expression of human cardiac R58Q-RLC (29, 30).

### Cloning, expression, and purification of recombinant RLC proteins

Reverse transcription-polymerase chain reaction and primers based on the published cDNA RLC sequence (GenBank™ Accession No. AF020768) were used to generate the cDNAs of human cardiac WT-RLC and RLC mutants. Standard methods described previously (5) were utilized to produce cDNAs of WT-RLC and two phosphomimetic RLC mutants, S15D (GenBank accession number ON950401) and T160D (GenBank accession number ON950400). Briefly, the cDNAs were obtained by overlapping sequential polymerase chain reaction and subsequently transformed into BL21 expression host cells to express proteins in 16 L cultures. Proteins were then purified by ion-exchange chromatography and eluted with a salt gradient of 0–450 mM NaCl. In the first step an S-Sepharose column was used equilibrated with 2 M urea, 20 mM sodium citrate, 0.1 mM PMSF, 1 mM 1,4-dithiothreitol (DTT), and 0.02% NaN_3_, pH 6.0. Eluted proteins were purified further using a Q-Sepharose column equilibrated with 2 M urea, 25 mM Tris–HCl, 0.1 mM PMSF, 1 mM DTT, and 0.02% NaN_3_, pH 7.5. The purity of all recombinant RLC proteins was determined by 15% SDS–PAGE (5, 21).

### Isolation and purification of mouse cardiac myosin

Cardiac myosin was isolated from mouse hearts of 9–12 month-old male and female Tg-WT and Tg-R58Q mice as described earlier (31). In short, myosin was extracted from the homogenized ventricular tissue in an ice-cold Guba Straub-type buffer (300 mM NaCl, 100 mM NaH_2_PO_4_, 50 mM Na_2_HPO_4_, 1 mM MgCl_2_, 10 mM EDTA, 0.1% NaN_3_, 10mM Na_4_P_2_O_7_, 1 mM DTT, and a protease inhibitor cocktail, pH 6.5). The extract was incubated on ice for 40 min and then ultracentrifuged at 200,000 g for 1 h (4°C). The supernatant was diluted 60-fold (by volume) with 2 mM DTT and left on ice for 60 min to precipitate filamentous myosin. Precipitated myosin was then centrifuged at 8,000 g for 10 min (4°C). The pellet was dissolved in a small volume of myosin buffer containing 0.4 M KCl, 10 mM 3-(n-morpholino) propane sulfonic acid (MOPS) (pH 7.0), 5 mM DTT, and a protease inhibitor cocktail. Myosin samples were diluted with glycerol in a 1:1 ratio and stored at −20°C until used. The purity of myosin preparations was tested by SDS-PAGE. Gel samples were prepared by mixing myosin solution 1:1 by volume with Laemmli buffer (62.5 mM Tris·HCl, pH 6.8, 25% glycerol, 2% SDS, 0.01% Bromophenol blue, and 5% β-mercaptoethanol, BME). Mixtures were heated at 95°C for 5 min and stored at −20°C until used for SDS-PAGE.

### Replacement of endogenous RLC from mouse cardiac myosin with the human cardiac WT and mutant RLCs

Depletion of endogenous RLC from mouse cardiac myosin (Tg-WT and Tg-R58Q) was achieved by using the buffer containing 1% Triton X-100 and 5 mM CDTA (cyclohexane-1,2- diamine tetra acetic acid), pH 8.5, as described earlier for porcine cardiac myosin (21). Reconstitution of depleted myosins was achieved by mixing them with a 3-fold molar excess of recombinant human cardiac RLC proteins, WT, S15D, T160D for Tg-WT myosin, and WT, S15D, T160D, and R58Q for Tg-R58Q myosin in a buffer containing 0.4 M KCl, 50 mM MOPS, pH 7.0, 2 mM MgCl_2_, and 1 mM DTT. After the brief incubation, the complexes were placed in dialyzing tubes and dialyzed against the same buffer for 2 h at 4°C. Subsequently, the complexes were transferred to 5 mM DTT and dialyzed overnight at 4°C. This process resulted in the precipitation of myosin reconstituted with different recombinant RLC proteins. Reconstituted myosin complexes were then centrifuged at 8,000 g for 10 min (4° C). Pelleted myosin-RLC complexes were resuspended in the buffer consisting of 0.4 M KCl, 10 mM MOPS, pH 7.0, and 1 mM DTT, mixed 1:1 with glycerol and stored at −20°C until needed. The quality of myosin-RLC complexes was tested by SDS–PAGE.

### ATPase measurements

Rabbit skeletal F-actin was used in the ATPase experiments. The protocol for actin purification was detailed in our earlier publication (21). Reconstituted mouse cardiac myosins (stored previously in 50% glycerol) were precipitated with 13 volumes of ice-cold 2 mM DTT and centrifuged at 8,000 g for 10 min (4°C). Pelleted myosins were resuspended in myosin buffer (0.4 M KCl, 10 mM MOPS, and 1 mM DTT, pH 7.0) and dialyzed against it overnight at 4°C. Following determination of the concentration of reconstituted myosin preparations with Coomassie Plus protein assay (Pierce, Rockford, IL, USA), myosin at 0.5 μM concentration was titrated with 0.1, 0.5, 1.5, 3, 5, 7.5, 10, and 15 μM F-actin. The assay was performed in duplicate on a 96-well plate in a buffer consisting of 25 mM imidazole (pH 7.0), 4 mM MgCl_2_, 1 mM EGTA, 1 mM DTT, and 77.7 mM final KCl salt concentration. The reaction, performed in a Jitterbug incubator shaker, was initiated by adding 2.5 mM ATP and continued for 15 min at 30°C. The reaction was terminated with 4% ice-cold trichloroacetic acid (TCA). Precipitated proteins were then centrifuged, and the supernatants were used for the assessment of inorganic phosphate by Fiske and Subbarow method (21, 32). Data points were fitted to Michaelis–Menten equation, yielding V_max_ (maximal activity) and K_m_ (Michaelis–Menten dissociation constant) (21, 33).

### Preparation of skinned LVPM fibers

Left ventricular papillary muscle (LVPM) fibers were isolated from 5 to 7 month-old Tg-WT and Tg-R58Q mice. They were dissected into small muscle bundles (~2–3 × 0.5–1 mm in size) in an ice-cold relaxing (pCa 8) solution (10^−8^ M Ca^2+^, 1 mM free Mg^2+^, total MgPr, propionate = 3.88 mM, 7 mM EGTA, 2.5 mM Mg-ATP^2−^, 20 mM MOPS pH 7.0, 15 mM creatine phosphate, and 15 U/ml of phosphocreatine kinase, ionic strength = 150 mM adjusted with KPr) and in the presence of 30 mM 2,3-Butanedione 2-monoxime (BDM) and 15% glycerol. Muscle bundles were then transferred to a fresh pCa 8 solution mixed with 50% glycerol (storage solution) and incubated for 1 h on ice. Muscle strips were chemically skinned in 1% Triton X-100 added to the mixture of pCa 8 solution and glycerol (50:50 by volume) overnight at 4°C. The bundles were transferred to a new storage solution and kept for 5–10 days at −20°C (30).

### CDTA-Extraction of endogenous RLC from LVPM fibers and reconstitution with recombinant RLC proteins

Endogenous RLC depletion from mouse LVPM preparations was achieved by treatment of fibers ~100 μm × ~1.5 mm in size isolated from glycerinated LVPM bundles with an extraction buffer composed of 5 mM CDTA, 1% Triton X-100, 50 mM KCl, 40 mM Tris, 0.6 mM NaN_3_, 0.2 mM PMSF at pH 8.4, and protease inhibitor cocktail for 40 min at room temperature. The CDTA/Triton-treated fibers were incubated with 15 μM of cardiac troponin C (TnC) in pCa 8 solution due to the potential extraction of endogenous TnC under these conditions. RLC-depleted and TnC reconstituted LVPM strips were subsequently incubated in pCa 8 solution containing 40 μM recombinant WT, T160D, or S15D RLC proteins for Tg-WT strips and 40 μM recombinant WT, R58Q, T160D, and S15D RLCs for Tg-R58Q strips for 45 min at room temperature. RLC and TnC reconstituted LVPM fibers were washed in a pCa 8 buffer and kept at −20°C for 1–5 days until needed for experiments. SDS-PAGE tested the degree of RLC depletion and RLC reconstitution in LVPM fibers.

### SDS-PAGE experiments

The myosin and LVPM samples were run on 15% SDS–PAGE gels. For myosin, ~20 μg of protein was used per well, and for LVPM, one fiber per well. The bands were stained with Coomassie Brilliant Blue. The gels were scanned using the Odyssey infrared imaging system (LICOR Biosciences, Lincoln, NE, USA). The level of RLC depletion and reconstitution was determined by densitometry analysis using ImageJ software (https://imagej.nih.gov/ij/) measuring RLC/ELC band intensities in native myosin/LVPM, RLC-depleted, and RLC-reconstituted myosin/LVPM. Myosin ELC that was not extracted during the RLC-depletion/reconstitution experiment was used as a loading control (34).

### Force-pCa study

LVPM fibers ~1.5 mm in length and ~100 μm in diameter were isolated from glycerinated muscle bundles, rinsed several times in pCa 8 solution, and mounted on the force transducer of the Guth Muscle Research System (Heidelberg, Germany). The fibers were treated with 1% Triton X-100 in pCa 8 buffer for 30 min at room temperature. After skinning with Triton X-100, muscle fibers were washed in pCa 8 buffer (3 times × 5 min), and their sarcomere length was adjusted to 2.1–2.2 μm. Then LVPM fibers were tested for maximal steady-state force development in pCa 4 solution, which has the same composition as pCa 8 buffer except the [Ca^2+^] = 10^−4^ M, and relaxed in pCa 8 solution. For the force–pCa relationship, the fibers were placed in solutions of increasing Ca^2+^ concentration from pCa 8 to pCa 4 and the level of force was measured in each “pCa” solution. The force-pCa dependence for RLC-depleted fibers was performed after the fibers were reconstituted with TnC. Maximal tension (in kN/m^2^) was determined from averaged values of tension measured before and after the force-pCa dependence and divided by the cross-sectional area of fibers. The diameter of fibers was estimated using an SZ6045 Olympus microscope with the measurement taken at 3 points along the fiber length and averaged. Force-pCa data were fitted to the Hill equation and the pCa_50_ (midpoint of force-pCa dependence and measure of calcium sensitivity) and n_H_ (Hill coefficient and measure of myofilament cooperativity) values were established for LVPM from Tg-WT, and Tg-R58Q mice reconstituted with recombinant RLCs proteins.

### Measurement of SRX ↔ DRX equilibrium by mant-ATP chase assay

Tg-WT and Tg-R58Q reconstituted LVPM fibers (~100 μm in diameter) were subjected to measurements of the super-relaxed (SRX) state of myosin. ATP turnover rates were measured by rapid exchange of fluorescent N-methylanthraniloyl (mant)-ATP with non-fluorescent (dark) ATP in skinned LVPM from all groups of mice using the previously described IonOptix Instrument (22, 35). The experiment was initiated with the fiber placed in a chamber under the microscope and incubated in a solution containing 250 μM mant-ATP in a rigor solution [120 mM KPr, 5 mM MgPr, 2.5 mM K_2_HPO_4_, 2.5 mM KH_2_PO_4_, 50 mM MOPS (pH 6.8), and fresh 2 mM DTT]. After fluorescence intensity reached a stable level, the fiber was chased with 4 mM non-labeled (dark) ATP. Fluorescence intensity decay isotherms were plotted as a function of time and fitted to a double-exponential equation Y = 1–P1[1-exp(–t/T1)]–P2[1–exp(–t/T2)], where P1 and P2 are the amplitudes of the fast phase, and slow phase of decay, respectively, and T1 and T2 are their respective lifetimes (36). To relate the P1 and P2 values to the number of myosin heads directly occupying the DRX and SRX states, the rapid phase of the fluorescence decay, P1, was corrected for the fast release of non-specifically bound mant-ATP. The correction was established experimentally using a competition assay and was equal to 0.44 ± 0.02, and the fraction of myosin heads directly occupying the SRX state was calculated as P2/(1–0.44) (35).

### Secondary structure prediction of WT and R58Q RLCs as well as their phosphomimetic mutants

The secondary structure prediction was executed using the I-TASSER approach developed by the Zhang laboratory, University of Michigan, and available online at http://zhanglab.ccmb.med.umich.edu/ITASSER/. The amino acid sequences of S15D/T160D phosphomimetic RLCs in the background of WT-RLC or R58Q-RLC were compared against template proteins of similar structures chosen from the protein data bank (PDB) library. High similarity structures were used: 5tbyE (Chain E, Myosin regulatory light chain 2, ventricular/cardiac muscle isoform), 3dtpE, 3jvtB (Chain B, calcium-bound Scallop Myosin Regulatory Domain (Lever Arm) with reconstituted complete Light Chains), 3pn7E, 3j04B (Chain B, EM structure of the heavy meromyosin subfragment of Chick smooth muscle myosin with regulatory light chain in phosphorylated state), 3i5iB, 2w4aB (Chain B, isometrically contracting Insect Asynchronous Flight Muscle), 2bl0C, 6iihA, 6k7yI. Fragments of the above templates were used for the assembly of the full-length protein, which was further simulated into the lowest-energy model using specific algorithms. Each predicted model structure was given a confidence C-score, ranging from −5 to 2, estimating the quality of the predicted models (37). The predicted protein structures were then visualized using PyMol (www.pymol.org).

### Statistical analysis

All values are shown as means ± SD with statistical significance determined as *p* < 0.05 using one-way ANOVA and Tukey's multiple comparison test (GraphPad Prism software version 7.0 for Windows).

## Results

To elucidate the effects of T160D and S15D phosphomimetic RLCs on cardiac muscle contraction and myosin energetics in HCM vs. WT hearts, we isolated cardiac myosin and LVPM preparations from Tg-R58Q and Tg-WT mouse models and subjected them to the depletion/reconstitution procedures. Reconstituted myosin and LVPM preparations were then used to measure actin-activated myosin ATPase activity, force-pCa relationship, and the super-relaxed state of myosin (36, 38). The latter is essential to understanding cardiac muscle's structure-function relationship and myosin energetic states (28).

### Effects of S15D and T160D phosphomimetic RLCs on myosin ATPase activity

**Figure 2:**
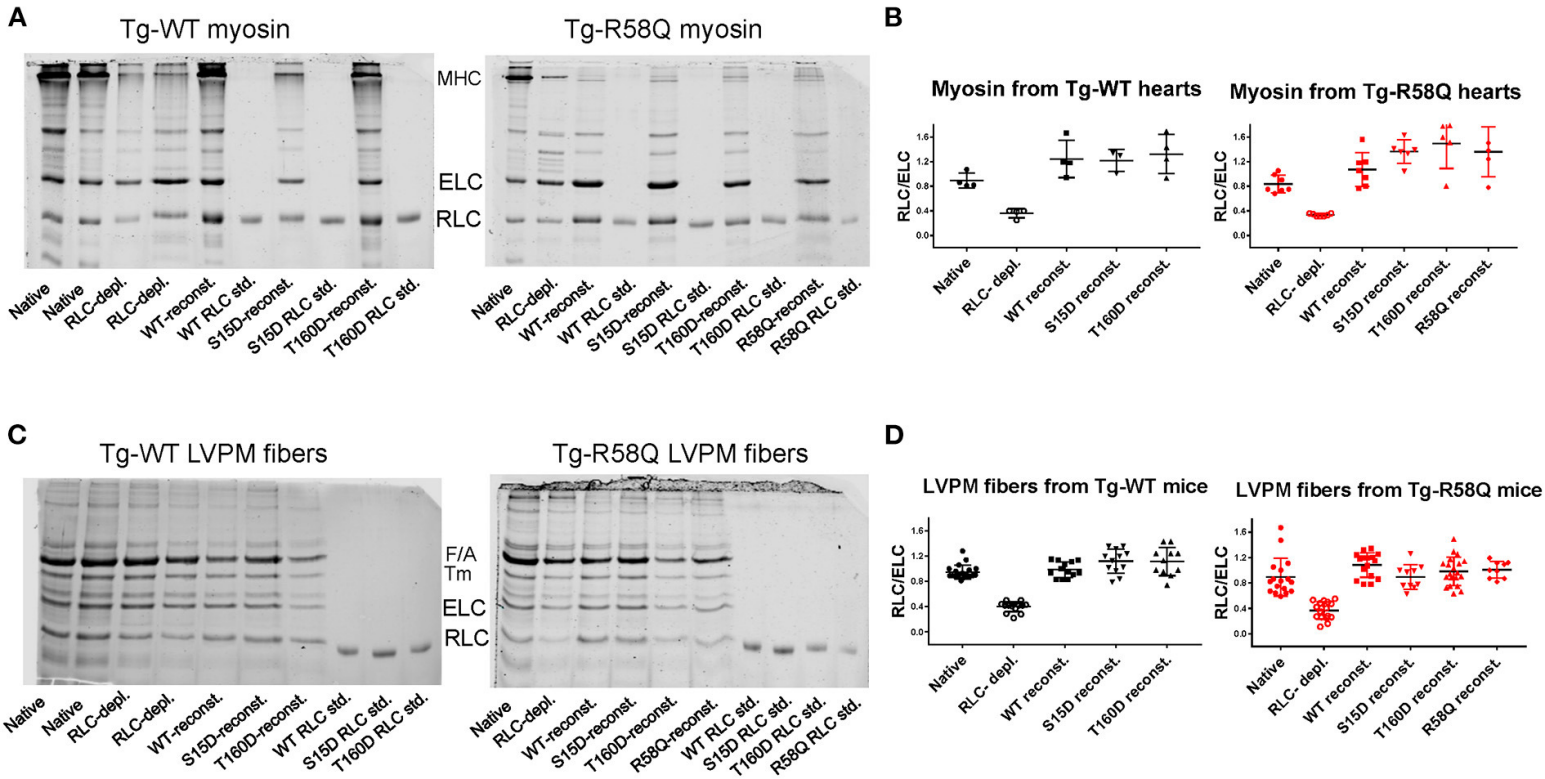
Representative SDS-PAGE images of RLC-depleted and mutant-reconstituted cardiac muscle preparations from Tg-WT and Tg-R58Q mice. **(A)** Depletion and reconstitution experiments in mouse-purified cardiac myosin. **(B)** Myosin gels' quantification. **(C)** Depletion and reconstitution experiments in LVPM fibers. **(D)** LVPM gels' quantification. Cardiac preparations were CDTA/Triton-depleted of endogenous RLC and reconstituted with recombinant WT-RLC, R58Q, T160D, and S15D RLC proteins. Three to Four different myosin preparations from Tg-WT and 5–7 from Tg-R58Q mice were used to deplete 60 ± 6% endogenous RLC, followed by myosin reconstitution with recombinant RLC proteins at 140 ± 30% in Tg-WT and 155 ± 40% Tg-R58Q myosin **(A,B)**. Eleven to Twenty LVPM fibers from Tg-WT and 9–20 from Tg-R58Q mice were employed to deplete 60 ± 12% RLC from LVPM, followed by 112 ± 20% reconstitution with recombinant RLC proteins in Tg-WT and 106 ± 21% in Tg-R58Q LVPM fibers **(C,D)**. Data in **(B,D)** are presented as means ± SD.

Myosin was extracted from heart ventricles of Tg-WT and Tg-R58Q mice and subjected to the depletion/reconstitution protocol, as described in Yadav et al. (22). The depletion of endogenous RLC resulted in ~40% of residual RLC still present in myosin from Tg-WT and Tg-R58Q hearts (Figure 2). The reconstitution of RLC-depleted Tg-WT or Tg-R58Q (Figure 2A) myosin with recombinant T160D, S15D, R58Q, and WT RLC proteins was achieved by incubation of RLC-depleted myosin with a 3-fold molar excess of recombinant RLC proteins. As shown in Figure 2B, RLC-depleted myosin was >100% reconstituted with recombinant RLC proteins, as judged by the RLC/ELC bands' ratios of SDS-PAGE. Reconstituted myosin preparations at a concentration of 0.5 μM were complexed with 0–15 μM actin, and the ATPase activity assays were performed.

**Figure 3:**
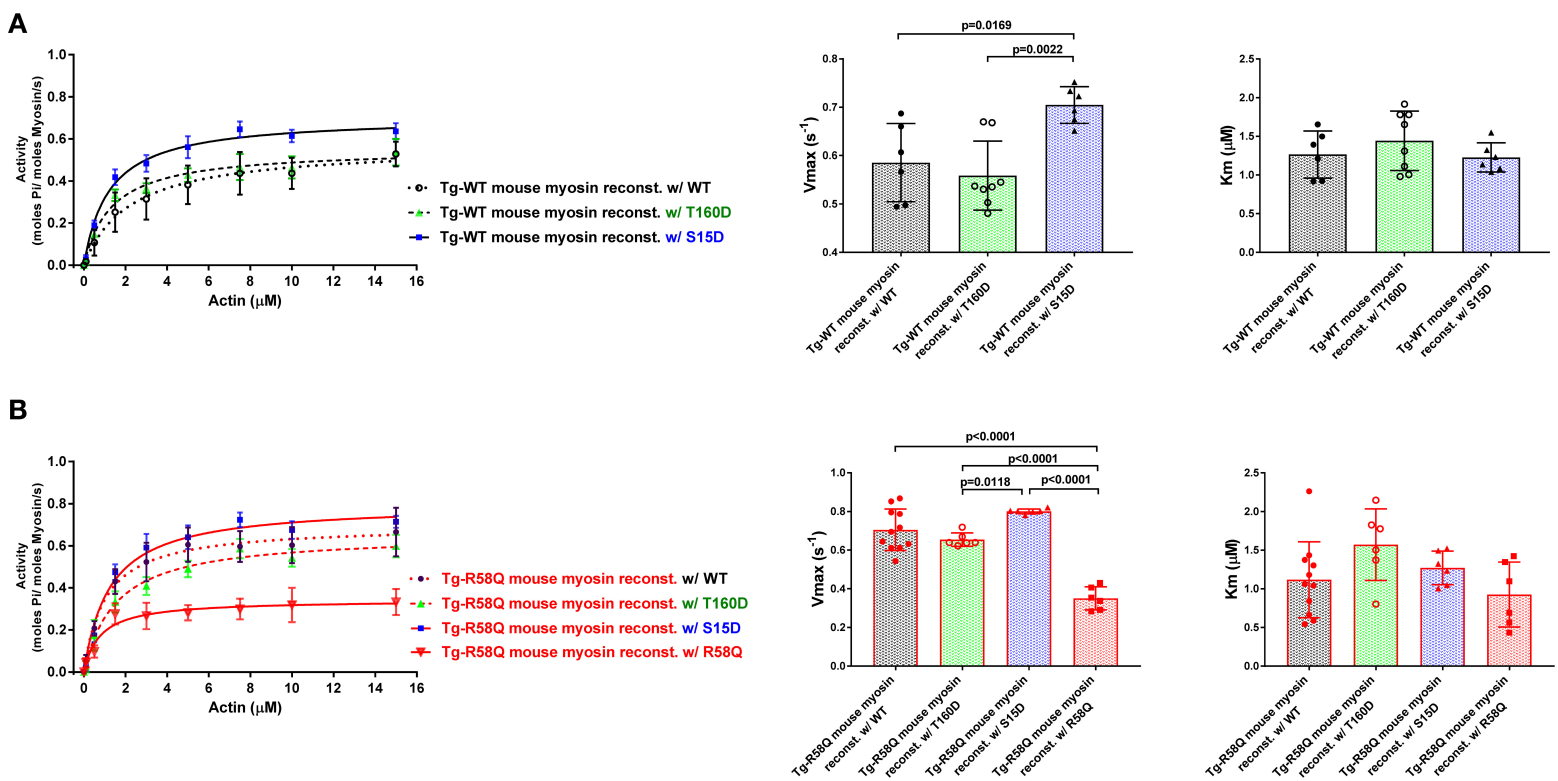
Summary of the actin-activated myosin ATPase activity study. Titration isotherms (left panels), and V_max_ (middle panels) and K_m_ (right panels) parameters of Tg-WT **(A)** and Tg-R58Q **(B)** myosin reconstituted with recombinant WT-RLC (black), T160D (green), S15D (blue), and R58Q (red) proteins. Myosin (0.5 μM) was complexed with actin of increasing concentrations (0–15 μM), and the actin-activated ATPase activity was determined for all reconstituted myosin preparations. Data are expressed as mean ± SD of *n* = N° experiments. For Tg-WT reconstituted w/WT-RLC, *n* = 6, w/T160D, *n* = 8, and w/S15D, *n* = 6. For Tg-R58Q reconstituted w/WT-RLC, *n* = 11, w/T160D, *n* = 6, w/S15D, *n* = 6, and w/R58Q, *n* = 6. Statistical significance between titration isotherms was calculated by two-way ANOVA with Tukey's multiple comparisons, with the *p*-values presented in Table 2. One-way ANOVA with Tukey's multiple comparisons was used to calculate significant differences in V_max_ and Km between the groups, and the *p*-values are presented in the graphs.

As shown in Figure 3A, the titration isotherms for T160D-RLC reconstituted Tg-WT myosin were similar to WT-RLC-reconstituted but significantly different than those of Tg-WT myosin reconstituted with S15D RLC. The V_max_ of S15D-RLC reconstituted Tg-WT myosin was 1.2 and 1.3-fold higher than that of WT or T160D-RLC reconstituted, respectively (Figure 3A; Table 1). The summary of statistical analysis of actin-activated myosin ATPase activity isotherms of Tg-WT and Tg-R58Q reconstituted myosin is presented in Table 2. Titration isotherms for HCM Tg-R58Q myosin reconstituted with WT-RLC, T160D, S15D, and R58Q are shown in Figure 3B and analyzed for significance in Table 2. For Tg-R58Q myosin, the V_max_ was the lowest in R58Q-RLC reconstituted compared with WT-RLC, T160D-RLC, or S15D-RLC-reconstituted (Figure 3B; Table 1). Both T160D and S15D phosphomimetic RLCs could restore the maximal ATPase activity in Tg-R58Q myosin to the level observed for WT-RLC reconstituted, but S15D-RLC appeared to be more effective, with significantly higher V_max_ compared to T160D-RLC-reconstituted Tg-R58Q myosin (Figure 3B; Table 1). Interestingly, in our previous study of actin-activated myosin ATPase activity measured in reconstituted porcine cardiac myosin, a significantly lower V_max_ of R58Q-RLC vs. WT-RLC reconstituted myosin could be rescued by the S15D phosphomimetic R58Q protein (22). In summary, significant differences were noted between S15D-RLC vs. T160D-RLC RLCs, with the S15D-RLC surpassing the T160D-RLC in recusing the low ATPase activity of R58Q-RLC-reconstituted myosin purified from HCM Tg-R58Q myocardium (Figure 3B; Tables 1, 2). The Km values were not significantly different among all tested proteins (Table 1).

**Table 1:** Summary of actin-activated myosin ATPase activity study.

**Parameter/recombinant RLC protein**	**Myosin from Tg-WT mice**			**Myosin from Tg-R58Q mice**			
	**WT**	**T160D**	**S15D**	**WT**	**T160D**	**S15D**	**R58Q**
V_max_ (s^−1^) ± SD	0.59 ± 0.08	0.56 ± 0.07^##^	0.71 ± 0.04*	0.71 ± 0.11	0.66 ± 0.04^#, ∧∧∧∧^	0.80 ± 0.01^∧∧∧∧^	0.35 ± 0.06****
K_m_ ± SD	1.27 ± 0.31	1.44 ± 0.38	1.23 ± 0.19	1.12 ± 0.49	1.57 ± 0.46	1.27 ± 0.22	0.93 ± 0.42
*n* = N° experiments	6	8	6	11	6	6	6

Values are means ± SD for n = N° of independent experiments. Significance was calculated by one-way ANOVA with Tukey's multiple comparison test with ^*^p <0.05 and ^****^p <0.0001 for S15D, T160D or R58Q for mutant vs. WT protein, ^#^p <0.05 and ^##^p <0.01 for T160D vs. S15D, ^∧∧∧∧^p <0.0001 for T160D and S15D vs. R58Q.

**Table 2:** Summary of statistical analysis of actin-activated myosin ATPase activity of Tg-WT and Tg-R58Q myosin reconstituted with recombinant human cardiac WT-RLC, S15D and T160D phosphomimetics, and R58Q mutant.

**System/actin concentration (μM)**	***p*** **-values**							
	**0.1**	**0.5**	**1.5**	**3**	**5**	**7.5**	**10**	**15**
Tg-WT reconst. w/WT vs. w/S15D	NS	NS	0.0037	0.0032	0.0002	<0.0001	<0.0001	0.0005
Tg-WT reconst. w/WT vs. w/T160D	NS	NS	NS	NS	NS	0.0459	NS	NS
Tg-WT reconst. w/T160D vs. w/S15D	NS	NS	0.0011	<0.0001	<0.0001	<0.0001	<0.0001	<0.0001
Tg-R58Q reconst. w/WT vs. w/R58Q	NS	0.001	<0.0001	<0.0001	<0.0001	<0.0001	<0.0001	<0.0001
Tg-R58Q reconst. w/WT vs. w/T160D	NS	NS	0.0056	0.0007	0.0006	NS	NS	NS
Tg-R58Q reconst. w/WT vs. w/S15D	NS	NS	NS	NS	NS	0.0001	NS	NS
Tg-R58Q reconst. w/T160D vs. w/S15D	NS	NS	0.0002	<0.0001	<0.0001	0.0003	0.0005	0.0033
Tg-R58Q reconst. w/T160D vs. w/R58Q	NS	NS	NS	0.0002	<0.0001	<0.0001	<0.0001	<0.0001
Tg-R58Q reconst. w/S15D vs. w/R58Q	NS	NS	<0.0001	<0.0001	<0.0001	<0.0001	<0.0001	<0.0001

Two-way ANOVA with Tukey's multiple comparison test was applied.

### Isometric force generation in mutant-reconstituted LVPM fibers from HCM-R58Q mice is rescued by S15D and T160D phosphomimetic RLC proteins

**Figure 4:**
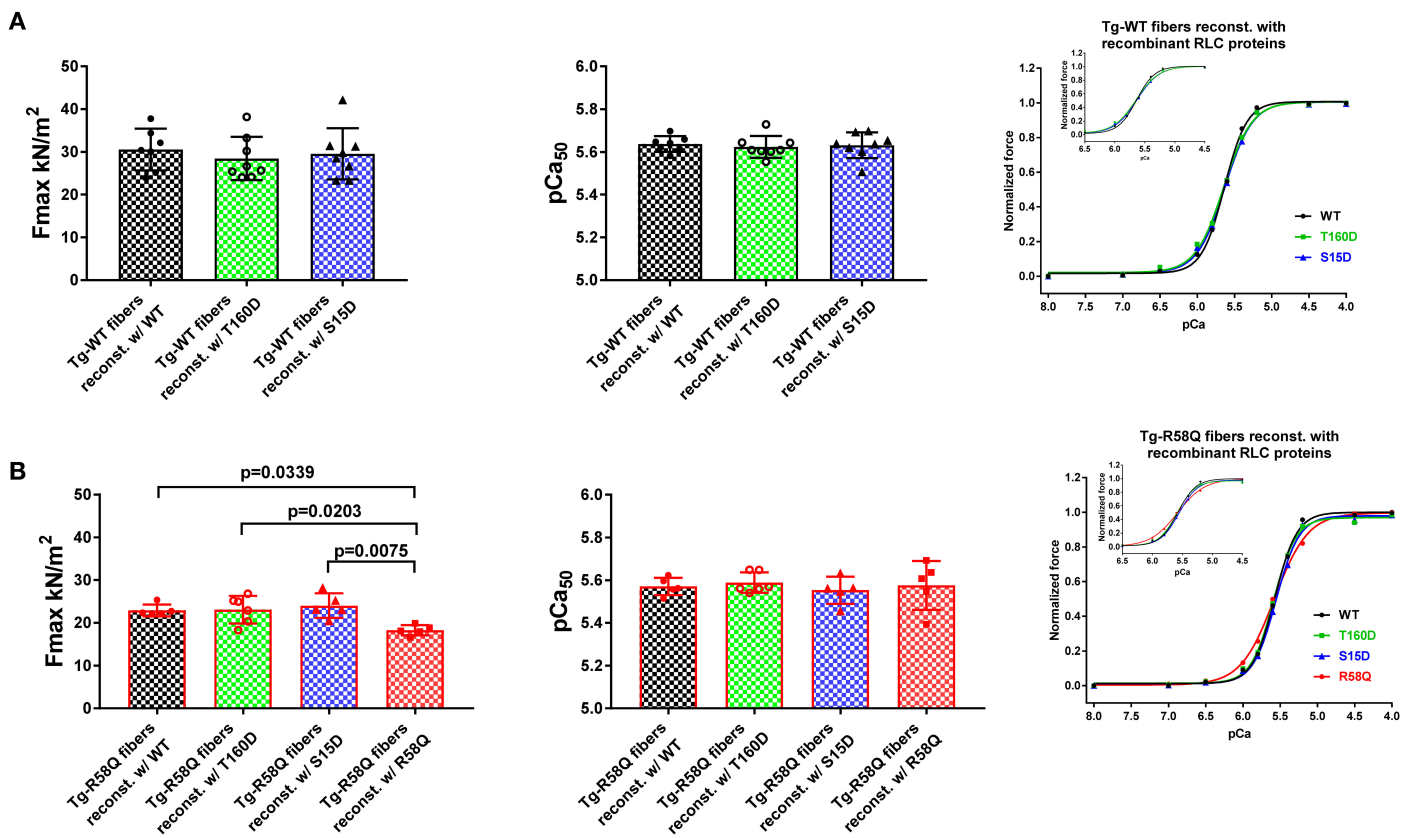
Contractile function in skinned LVPM from Tg-WT **(A)** and Tg-R58Q **(B)** mice reconstituted with recombinant RLC proteins. Maximal force (left panels), Calcium sensitivity of force (middle panels), and force-pCa relationships (right panels) were measured in LVPM from Tg-WT and Tg-R58Q preparations depleted of endogenous RLC and reconstituted with recombinant WT-RLC (black), T160D (green), S15D (blue), and R58Q (red) proteins. For Tg-WT fibers reconstituted w/WT, *n* = 7 fibers, w/T160D, *n* = 8, and w/S15D, *n* = 8, and Tg-R58Q reconstituted w/WT, *n* = 5, w/T160D, *n* = 6, w/S15D, *n* = 5, and w/R58Q, *n* = 5. Data are the average ± SD of *n* = N° fibers per group with the significance calculated by one-way ANOVA with Tukey's multiple comparison test (for F_max_ and pCa50 values, *p*-values presented in the graph), or two-way ANOVA with Tukey's multiple comparison test (for the force-pCa relationships).

The effects of RLC phosphomimetics on the force-pCa dependence were assessed in skinned LVPM fibers from Tg-WT and HCM Tg-R58Q mice that were depleted of endogenous RLC protein and reconstituted with either S15D or T160D RLCs along with WT and R58Q RLC controls (Figures 4A,B). Representative images of CDTA/Triton-depleted and RLC/TnC-reconstituted mouse LVPM from Tg-WT and Tg-R58Q hearts are presented in Figure 2C. As illustrated in Figure 2D, ~60% of RLC depletion could be achieved in Tg-WT and Tg-R58Q LVPM fibers that were subsequently ~110% reconstituted with recombinant RLC proteins (Figure 2D).

In Tg-WT, maximum isometric tension per cross-section of muscle (in kN/m^2^) and force-pCa relationship were similar among all reconstituted fibers (Figure 4A; Table 3). However, LVPM fibers from Tg-R58Q show the lowest level of force for R58Q-reconstituted compared with S15D, T160D, and WT-RLC-reconstituted LVPM (Figure 4B). Both S15D and T160D phosphomimetic RLC mutants were equally efficient in bringing up the level of force to that observed for WT-RLC-reconstituted fibers from Tg-R58Q mice (Figure 4B; Table 3). Like for LVPM from Tg-WT mice, the calcium sensitivity of force, represented by pCa_50_, was not different among all tested RLC proteins reconstituted in Tg-R58Q fibers (Figure 4; Table 3). Differences were noted in the Hill coefficient (n_H_) between WT and mutant-reconstituted fibers from both groups of transgenic fibers (Table 3).

**Table 3:** Contractile function in skinned LVPM from Tg-WT and Tg-R58Q mice reconstituted with recombinant RLCs proteins.

**Parameter/recombinant RLC protein**	**LVPM from Tg-WT mice**			**LVPM from Tg-R58Q mice**			
	**WT**	**T160D**	**S15D**	**WT**	**T160D**	**S15D**	**R58Q**
Fmax (kN/m^2^) ± SD	30.54 ± 4.9	28.44 ± 5.07	29.57 ± 6.01	22.92 ± 1.39^∧^	23.09 ± 3.22^∧^	24.04 ± 1.3^∧∧^	18.28 ± 1.22
pCa_50_ ± SD	5.64 ± 0.04	5.62 ± 0.05	5.63 ± 0.06	5.57 ± 0.04	5.59 ± 0.05	5.55 ± 0.06	5.58 ± 0.11
n_H_ Hill coeff. ± SD	3.01 ± 0.58	2.45 ± 0.42	2.41 ± 0.33*	2.98 ± 0.3	2.9 ± 0.23^∧∧∧∧^	2.9 ± 0.19^∧∧∧^	2 ± 0.28****
*n* = N° fibers	7	8	8	5	6	5	5

Values are means ± SD for n = N° of independent experiments. Significance was calculated by one-way ANOVA with Tukey's multiple comparison test with *p <0.05 and ****p <0.0001 for S15D, T160D or R58Q mutant vs. WT protein, ^∧^p <0.05, ^∧∧^p <0.01, ^∧∧∧^p = 0.001, and ^∧∧∧∧^p <0.0001 for S15D, T160D, or WT vs. R58Q.

### Mutant RLC-mediated regulation of SRX↔DRX equilibrium in LVPM from HCM Tg-R58Q vs. Tg-WT mice

**Figure 5:**
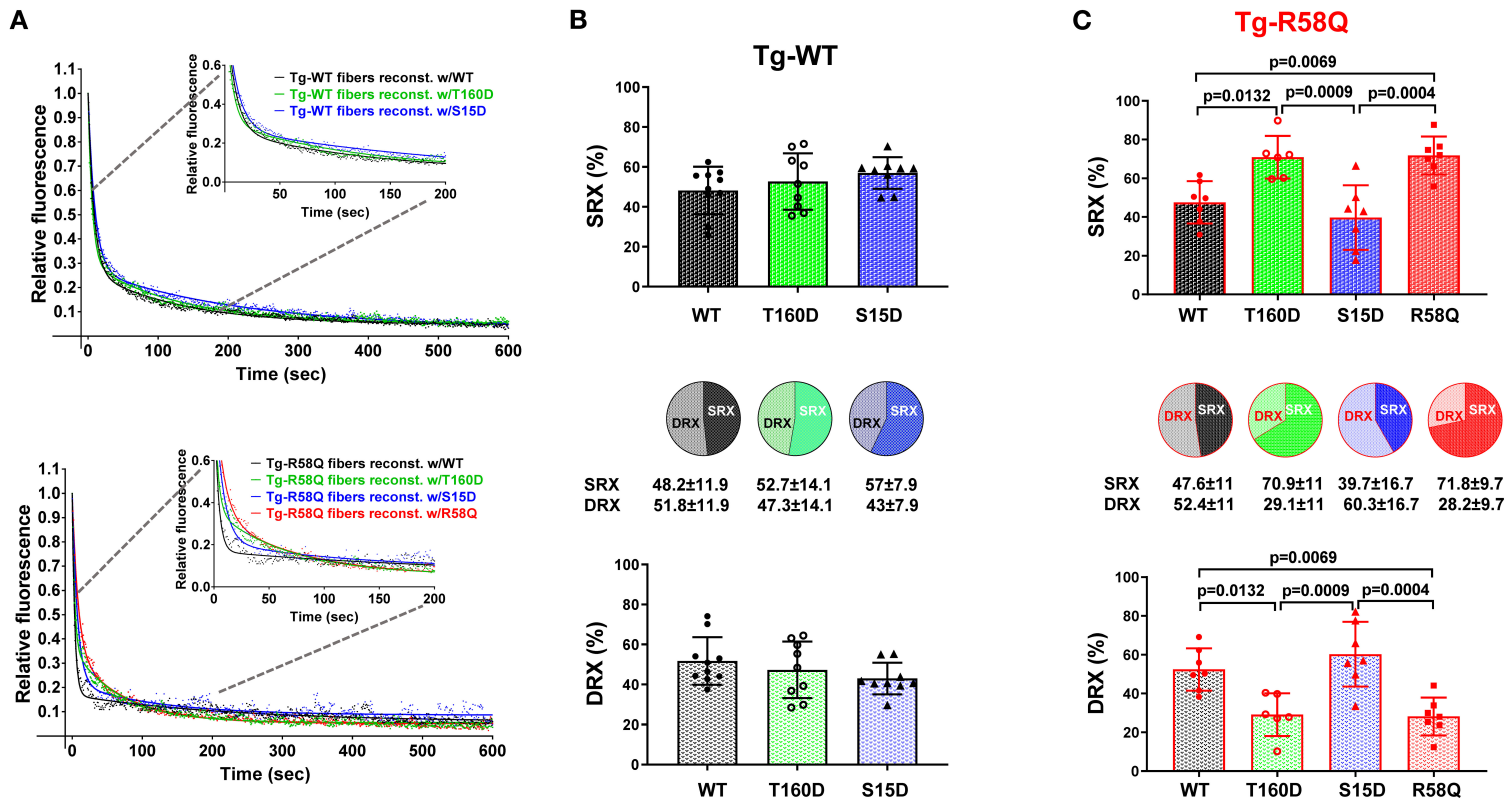
Summary of the super-relaxed (SRX) state study. **(A)** Fluorescence decay curves in RLC-depleted and mutant-reconstituted LVPM fibers from Tg-WT (upper panel) and Tg-R58Q (lower panel) hearts. **(B)** Distribution of myosin heads between the SRX and DRX states in Tg-WT fibers reconstituted w/WT-RLC (black), w/T160D (green), and w/S15D (blue). **(C)** Distribution of myosin heads between the SRX and DRX states in Tg-R58Q fibers reconstituted w/WT (black), w/T160D (green), w/S15D (blue), and w/R58Q (red). Data are the average ± SD of n fibers per group. For Tg-WT fibers reconstituted w/WT, *n* = 10, w/T160D, *n* = 9, and w/S15D, *n* = 9. For Tg-R58Q reconstituted w/WT, *n* = 7, w/T160D, *n* = 6, w/S15D, *n* = 7, and w/R58Q, *n* = 7. The significance was calculated by one-way ANOVA with Tukey's multiple comparison test with p values depicted in the graph. Note that the % SRX is increased in R58Q reconstituted Tg-R58Q fibers.

In contracting muscle, myosin cross-bridges oscillate between the active and relaxed states, with the latter comprising the SRX energy-saving conformation and the DRX state facilitating cross-bridge formation with greater ATP consumption (39). Under relaxation conditions, myosin cross-bridges may exist in various structural and biochemical states, and each state is associated with different energy consumption rates (40). Modulation of myosin function through the SRX mechanism is essential for sarcomere contraction, and many factors, e.g., mutations in sarcomeric proteins, may affect SRX ↔ DRX equilibrium. To further explore the differences and similarities between the two RLC phosphorylation sites at S15 and T160, we assessed the effect of phosphomimetic RLC mutants on the SRX state and SRX ↔ DRX equilibrium following their exchange for the endogenous cardiac RLC in LVPM from Tg-WT and HCM Tg-R58Q mice (Figure 5; Table 4). LVPM fibers underwent the RLC-depletion/reconstitution procedure and then were subjected to mant-ATP chase assay (22, 35). The fluorescence decay curves vs. time were collected on the rapid exchange of fluorescent mant-ATP for non-labeled (dark) ATP (Figure 5A). The data were fitted to a two-state exponential equation, and the amplitudes of the fast (P1) and slow (P2) phases of fluorescence decay and their respective T1 and T2 lifetimes (in seconds) were obtained (22, 35, 41). To estimate the number of myosin heads directly occupying the SRX state in reconstituted LVPM fibers from Tg-WT (Figure 5B) and Tg-R58Q hearts (Figure 5C), the rapid phase of the fluorescence decay (P1) was corrected for the fast release of nonspecifically bound mant-ATP and the number of SRX heads calculated as P2/(1–0.44) (35). No differences in the SRX-to-DRX ratio were observed for the mutant RLC-reconstituted LVPM from Tg-WT (Figure 5B; Table 4). However, assessment of mutant RLC-reconstituted LVPM fibers from Tg-R58Q showed a significantly higher proportion of myosin cross-bridges in the SRX state for fibers reconstituted with recombinant R58Q-RLC (~72%) compared with WT-RLC-reconstituted fibers (48%). This result supports our previous data on R58Q-RLC reconstituted porcine and mouse cardiac preparations showing that R58Q promotes the OFF state of myosin by stabilizing the SRX conformation characterized by a very low ATP turnover rate (22). Interestingly, S15D-RLC destabilized the SRX state and shifted the R58Q heads toward the DRX state (Figure 5C; Table 4). The T160D phosphomimetic RLC did not alter the SRX-to-DRX ratio in Tg-R58Q LVPM fibers and behaved similarly to fibers reconstituted with R58Q-RLC (Figure 5C; Table 4). No significant differences in the lifetimes of fast and slow phases of fluorescence decays curves were observed among all tested systems (Table 4). As for the ATPase assay shown in Figure 3, a significant difference was noted between S15D-RLC vs. T160D-RLC RLCs, with the S15D-RLC being superior to T160D-RLC in recusing the hypocontractile behavior of R58Q-RLC-reconstituted fibers from HCM Tg-R58Q myocardium (Figure 5C).

**Table 4:** The SRX state of myosin measured by mant-ATP/ATP chase assays in skinned LVPM from Tg-WT and Tg-R58Q mice reconstituted with recombinant RLCs proteins.

**Parameter/recombinant RLC protein**	**LVPM from Tg-WT mice**			**LVPM from Tg-R58Q mice**			
	**WT**	**T160D**	**S15D**	**WT**	**T160D**	**S15D**	**R58Q**
DRX ± SD (%)	51.8 ± 11.9	47.3 ± 14.1	43 ± 7.9	52.4 ± 11	29.1 ± 11*^, *###*^	60.3 ± 16.7^∧∧∧^	28.2 ± 9.7**
SRX ± SD (%)	48.2 ± 11.9	52.7 ± 14.1	57 ± 7.9	47.6 ± 11	70.9 ± 11*^, *###*^	39.7 ± 16.7^∧∧∧^	71.8 ± 9.7**
T1 ± SD (s)	9.5 ± 6	5.9 ± 3	8.8 ± 3.9	3.9 ± 2.8	3.3 ± 2.5	7.8 ± 4.1	9 ± 8
T2 ± SD (s)	133.7 ± 93	111.5 ± 66.8	327.5 ± 313.7	107.4 ± 143.5	80 ± 85.9	225 ± 158.3	108.5 ± 158.2
*n* = N° fibers	10	9	9	7	6	7	7

Values are means ± SD for n = N° of independent experiments. Significance was calculated by one-way ANOVA with Tukey's multiple comparison test with *p <0.05 and **p <0.01 for R58Q, T160D mutant vs. WT protein, ^###^p <0.001 for T160D vs. S15D, ^∧∧∧^p <0.001 for S15D vs. R58Q.

## Discussion

Human cardiac RLC contains a cardiac MLCK (*MYLK3* gene)-specific phosphorylation site at serine-15 (S15) recognized by many research studies as being essential for heart performance in normal and disease conditions (42). Significantly decreased phosphorylation of the RLC occurs in heart failure patients (43–45) and is also observed in animal models of heart disease (23, 46, 47). The myocardium containing dephosphorylated myosin has a reduced ability to generate force and sustain cardiac function at steady-state levels (23, 48), suggesting that RLC phosphorylation may inspire the development of target-specific new therapies. Studies from our laboratory identified a link between compromised RLC phosphorylation in animal models of HCM and decreased force generation (48–51). Our *in vitro* data suggested that S15D phosphomimetic RLC where S15 is replaced by aspartic acid (D15), could serve as a strategy to mitigate the adverse cardiac phenotypes *in vivo*. Beneficial effects of S15D were observed in S15D-D166V transgenic mice, where the expression of S15D in the background of HCM-D166V mutation prevented the development of hypertrophy and cardiac dysfunction associated with D166V (23). The effects of S15D phosphomimetic RLC protein were recently tested *in vivo* when the S15D-RLC molecule was delivered into the hearts of Tg-D166V mice *via* the adeno-associated virus AAV9 (24). We observed a significant improvement in heart function in AAV9-S15D-RLC injected hearts of HCM mice compared with empty vector/PBS injected hearts (24).

Our *in-silico* search for other potential phosphorylation sites in the human RLC brought about several Ser/Thr/Tyr sites (Figure 1), of which we chose to focus on threonine-160 (T160). This is because T160 is located in the very C-terminal region of the RLC that encompasses a hot spot for HCM-associated mutations in the *MYL2* gene (17–20). As phosphorylation sites of myosin RLC may represent a potential target for therapeutic interventions, we tested whether S15D and T160D phosphomimetic RLCs can rescue cardiomyopathy phenotypes in a mouse model of HCM, Tg-R58Q mice (29). This approach has been previously tested in S15D phosphomimetic RLC-reconstituted cardiac preparations from HCM-D166V and HCM-R58Q mice, where S15D alleviated some of the detrimental HCM phenotypes *in vitro* (21, 22).

In this study, we compared the T160 RLC site with the established S15-RLC site and performed a series of reconstitution experiments using RLC-depleted myosin and LVPM fibers that were reconstituted with phosphomimetic T160D and S15D RLC proteins. The data demonstrated that when reconstituted in cardiac myosin and tested for actin-activated myosin ATPase activity, both phosphomimetic RLC proteins (T160D and S15D) were able to restore the maximal ATPase activity (V_max_) in Tg-R58Q myosin to the level of WT-RLC-reconstituted HCM-R58Q myosin (Figure 3B; Table 1). A significant difference in V_max_ was noted between S15D-RLC and T160D-RLC, with S15D-RLC showing higher ATPase than T160D reconstituted in Tg-WT and Tg-R58Q myosin, indicating functional superiority of the S15D vs. T160D phosphomimetic RLC protein. Significant differences were observed between both phosphomimetic RLCs in regulating SRX↔DRX equilibrium in Tg-R58Q mice (Figure 5). Unlike S15D-RLC, T160D-RLC did not change the ratio of the SRX to DRX state in skinned LVPM fibers of HCM Tg-R58Q mice. At the same time, S15D-RLC fostered the transition from the energy-conserving SRX state to the DRX state and increased the number of DRX heads readily available to interact with actin and produce force (Figure 5). No changes were noted in SRX ↔ DRX equilibrium by S15D or T160D RLCs in Tg-WT mice, indicating that the phosphorylation of S15-RLC is essential for rescuing the energetic state of myosin altered by HCM-R58Q mutation of the RLC.

Several studies suggest that the primary effect of HCM-causing mutations is hypercontractility of the heart that results from an increase in the number of functionally accessible myosin heads for the interaction with thin filaments and force production (52). The R58Q model displays a non-canonical HCM phenotype that is hypo- rather than hypercontractile (Figure 5). It stabilizes the OFF state of myosin in LVPM fibers from Tg-R58Q mice (Table 4; Figure 5) and in R58Q recombinant protein-reconstituted porcine fibers (22). As demonstrated by Kampourakis et al. (25), R58Q promotes the OFF state by reducing the number of myosin cross-bridges readily available for actin interaction and ATP utilization. Altogether, our previous and current results suggest that the abnormal heart performance in Tg-R58Q mice (29, 49) originates from an R58Q-mediated decrease in RLC phosphorylation, diminished maximal tension, and stabilization of the hypocontractile SRX state of myosin cross-bridges. These adverse HCM phenotypes can be rescued in full by the S15D and to some degree by the T160D phosphomimetic mutant.

**Figure 6:**
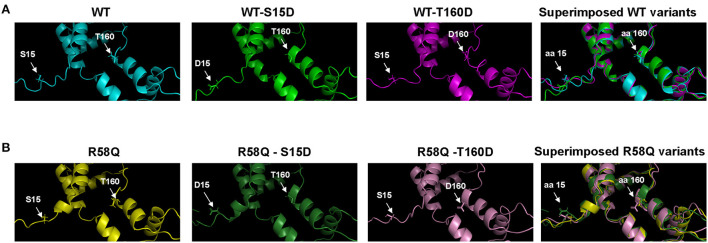
The S15 and T160 phosphorylation sites are visualized as phosphomimetics S15D and T160D in the human cardiac RLC-WT **(A)** and RLC-R58Q **(B)** proteins. I-TASSER derived secondary structures of the human cardiac RLC were built using protein templates selected from the Protein Data Bank (PDB): 5tbyE, 3dtpE, 3jvtB, 3pn7E, 3j04B, 3i5iB, 2w4aB, 2bl0C, 6iihA, and 6k7yI. Note that mild intramolecular rearrangements in the vicinity of phosphorylation sites at amino acids (aa) 15 and 160 are visible in the superimposed structures of WT and R58Q proteins.

The I-TASSER/PyMol modeled secondary structures of the phosphomimetic S15D and T160D mutants in the background of either WT-RLC or HCM R58Q-RLC are presented in Figure 6. The S15D mutation causes slight conformational changes in the N-terminus of WT-RLC (Figure 6A) and HCM R58Q-RLC (Figure 6B), indicating phosphorylation-mediated intramolecular changes in the RLC molecule. The T160D mutation appears to render more structural changes in the WT-RLC background (Figure 6A) than in the R58Q-RLC (Figure 6B), supporting its lesser rescue ability of function in HCM myocardium compared with S15D-RLC.

## Conclusions

Our experimental approach allowed us to test the functional significance of two phosphomimetic RLCs when reconstituted in cardiac muscle preparations from HCM-R58Q myocardium compared with WT mice. We showed that several R58Q-exerted adverse phenotypes could be rescued by S15D or T160D phosphomimetic RLCs in cardiac preparations from Tg-R58Q mice. A low level of maximal isometric force or depressed ATPase activity observed for R58Q-reconstituted samples could be restored by both S15D and T160D RLCs with the significantly better rescue of the V_max_ of myosin ATPase activity by S15D-RLC. Significantly, S15D but not T160D phosphomimetic RLC could modulate myosin energetic states in the resting muscle and promote the DRX state reducing the fraction of SRX heads and counteracting the hypocontractile activity of R58Q-reconstituted HCM myocardium. This result supports the functional superiority of the established serine-15 phosphorylation site of the RLC that may serve as a therapeutic target for HCM.

## Data availability statement

The original contributions presented in the study are publicly available. This data can be found at: https://www.ncbi.nlm.nih.gov/genbank/, BankIt2600965 T160D ON950400 and BankIt2600993 S15D ON950401.

## Ethics statement

The animal study was reviewed and approved by this study conforms to the Guide for the Care and Use of Laboratory Animals published by the US National Institutes of Health (NIH Publication No. 85–23, revised 2011). All protocols were approved by the Institutional Animal Care and Use Committee at the University of Miami Miller School of Medicine (protocol #21-106 LF). The assurance number is #A-3224-01, approved through November 30, 2023. Euthanasia of mice was achieved through inhalation of isoflurane followed by thoracotomy.

## Author contributions

KK and DS-C conceived the research and wrote the paper. KK, JL, and MG-G performed experiments. All authors contributed to the article and approved the submitted version.

## Funding

This study was supported by the National Institutes of Health (Grant Nos. R01-HL143830 and R56-HL146133 to DS-C).

## Conflict of interest

The authors declare that the research was conducted in the absence of any commercial or financial relationships that could be construed as a potential conflict of interest.

## Publisher's note

All claims expressed in this article are solely those of the authors and do not necessarily represent those of their affiliated organizations, or those of the publisher, the editors and the reviewers. Any product that may be evaluated in this article, or claim that may be made by its manufacturer, is not guaranteed or endorsed by the publisher.

## Abbreviations

DRX: Disordered relaxed state
ELC: Essential light chain of myosin
HCM: Hypertrophic cardiomyopathy
Mant-ATP: (*N*-Methylanthraniloyl) -ATP (fluorescent)
MHC: Myosin heavy chain
MLCK: Myosin light chain kinase
n_H_: Hill coefficient
P1: Amplitude of fast DRX phase
P2: Amplitude of slow SRX phase
R58Q: Arg → Gln mutation in myosin RLC
RLC: Regulatory light chain of myosin
S15D: Phosphomimetic RLC where serine-15 is replaced by aspartic acid
SRX: Super-relaxed state
T1: Nucleotide turnover lifetime in DRX
T2: Nucleotide turnover lifetime in SRX
T160D: Phosphomimetic RLC where threonine 160 is replaced by aspartic acid
Tg: Transgenic
WT: Wild-type.

## References

[1] Rayment I Rypniewski WR Schmidt-Base K Smith R Tomchick DR Benning MM. Three-dimensional structure of myosin subfragment-1: a molecular motor. Science. (1993) 261:50–8. 10.1126/science.8316857 8316857

[2] Geeves MA. Molecular motors: stretching the lever-arm theory. Nature. (2002) 415:129–31. 10.1038/415129a 11805818

[3] Burghardt TP Josephson MP Ajtai K. Single myosin cross-bridge orientation in cardiac papillary muscle detects lever-arm shear strain in transduction. Biochemistry. (2011) 50:7809–21. 10.1021/bi2008992 21819137 PMC3177300

[4] Burghardt TP Sikkink LA. Regulatory light chain mutants linked to heart disease modify the cardiac myosin lever arm. Biochemistry. (2013) 52:1249–59. 10.1021/bi301500d 23343568 PMC3587134

[5] Szczesna D Ghosh D Li Q Gomes AV Guzman G Arana C. Familial hypertrophic cardiomyopathy mutations in the regulatory light chains of myosin affect their structure, Ca^2+^ binding, and phosphorylation. J Biol Chem. (2001) 276:7086–92. 10.1074/jbc.M009823200 11102452

[6] Szczesna-Cordary D. Regulatory light chains of striated muscle myosin. Structure, function and malfunction. Curr Drug Targets Cardiovasc Haematol Disord. (2003) 3:187–97. 10.2174/1568006033481474 12769642

[7] Chang AN Battiprolu PK Cowley PM Chen G Gerard RD Pinto JR. Constitutive phosphorylation of cardiac myosin regulatory light chain in vivo. J Biol Chem. (2015) 290:10703–16. 10.1074/jbc.M115.642165 25733667 PMC4409237

[8] Chang AN Mahajan P Knapp S Barton H Sweeney HL Kamm KE. Cardiac myosin light chain is phosphorylated by Ca2+/calmodulin-dependent and -independent kinase activities. Proc Natl Acad Sci USA. (2016) 113:E3824–33. 10.1073/pnas.1600633113 27325775 PMC4941474

[9] Colson BA Locher MR Bekyarova T Patel JR Fitzsimons DP Irving TC. Differential roles of regulatory light chain and myosin binding protein-C phosphorylations in the modulation of cardiac force development. J Physiol. (2010) 588 (Pt. 6):981–93. 10.1113/jphysiol.2009.183897 20123786 PMC2849963

[10] Kampourakis T Irving M. Phosphorylation of myosin regulatory light chain controls myosin head conformation in cardiac muscle. J Mol Cell Cardiol. (2015) 85:199–206. 10.1016/j.yjmcc.2015.06.002 26057075 PMC4535163

[11] Wang Y Ajtai K Burghardt TP. Ventricular myosin modifies in vitro step-size when phosphorylated. J Mol Cell Cardiol. (2014) 72:231–7. 10.1016/j.yjmcc.2014.03.022 24726887 PMC4037356

[12] Yu H Chakravorty S Song W Ferenczi MA. Phosphorylation of the regulatory light chain of myosin in striated muscle: methodological perspectives. Eur Biophys J. (2016) 45:779–805. 10.1007/s00249-016-1128-z 27084718 PMC5101276

[13] Kampourakis T Sun YB Irving M. Myosin light chain phosphorylation enhances contraction of heart muscle via structural changes in both thick and thin filaments. Proc Natl Acad Sci USA. (2016) 113:E3039–47. 10.1073/pnas.1602776113 27162358 PMC4889392

[14] Scruggs SB Reisdorph R Armstrong ML Warren CM Reisdorph N Solaro RJ. A novel, in-solution separation of endogenous cardiac sarcomeric proteins and identification of distinct charged variants of regulatory light chain. Mol Cell Proteomics. (2010) 9:1804–18. 10.1074/mcp.M110.000075 20445002 PMC2938104

[15] Blom N Sicheritz-Pontén T Gupta R Gammeltoft S Brunak S. Prediction of post-translational glycosylation and phosphorylation of proteins from the amino acid sequence. Proteomics. (2004) 4:1633–49. 10.1002/pmic.200300771 15174133

[16] Dou Y Yao B Zhang C. PhosphoSVM: prediction of phosphorylation sites by integrating various protein sequence attributes with a support vector machine. Amino Acids. (2014) 46:1459–69. 10.1007/s00726-014-1711-5 24623121

[17] Richard P Charron P Carrier L Ledeuil C Cheav T Pichereau C. Hypertrophic cardiomyopathy: distribution of disease genes, spectrum of mutations, and implications for a molecular diagnosis strategy. Circulation. (2003) 107:2227–32. 10.1161/01.CIR.0000066323.15244.54 12707239

[18] Alvarez-Acosta L Mazzanti A Fernández X Ortí M Barriales-Villa R García D. Regulatory light chain (MYL2) mutations in familial hypertrophic cardiomyopathy. JCVD. (2014) 2:82–90.9535554

[19] De Bortoli M Vio R Basso C Calore M Minervini G Angelini A. Novel missense variant in MYL2 gene associated with hypertrophic cardiomyopathy showing high incidence of restrictive physiology. Circ Genom Precis Med. (2020) 13:e002824. 10.1161/CIRCGEN.119.002824 32004434

[20] Manivannan SN Darouich S Masmoudi A Gordon D Zender G Han Z. Novel frameshift variant in MYL2 reveals molecular differences between dominant and recessive forms of hypertrophic cardiomyopathy. PLOS Genet. (2020) 16:e1008639. 10.1371/journal.pgen.1008639 32453731 PMC7274480

[21] Muthu P Liang J Schmidt W Moore JR Szczesna-Cordary D. *In vitro* rescue study of a malignant familial hypertrophic cardiomyopathy phenotype by pseudo-phosphorylation of myosin regulatory light chain. Arch Biochem Biophys. (2014) 552–3:29–39. 10.1016/j.abb.2013.12.011 24374283 PMC4043912

[22] Yadav S Kazmierczak K Liang J Sitbon YH Szczesna-Cordary D. Phosphomimetic-mediated in vitro rescue of hypertrophic cardiomyopathy linked to R58Q mutation in myosin regulatory light chain. FEBS J. (2019) 286:151–68. 10.1111/febs.14702 30430732 PMC6326841

[23] Yuan CC Muthu P Kazmierczak K Liang J Huang W Irving TC. Constitutive phosphorylation of cardiac myosin regulatory light chain prevents development of hypertrophic cardiomyopathy in mice. Proc Natl Acad Sci USA. (2015) 112:E4138–46. 10.1073/pnas.1505819112 26124132 PMC4522794

[24] Yadav S Yuan CC Kazmierczak K Liang J Huang W Takeuchi LM. Therapeutic potential of AAV9-S15D-RLC gene delivery in humanized MYL2 mouse model of HCM. J Mol Med. (2019) 97:1033–47. 10.1007/s00109-019-01791-z 31101927 PMC6584042

[25] Kampourakis T Ponnam S Irving M. Hypertrophic cardiomyopathy mutation R58Q in the myosin regulatory light chain perturbs thick filament-based regulation in cardiac muscle. J Mol Cell Cardiol. (2018) 117:72–81. 10.1016/j.yjmcc.2018.02.009 29452157 PMC5883317

[26] Karabina A Kazmierczak K Szczesna-Cordary D Moore JR. Myosin regulatory light chain phosphorylation enhances cardiac beta-myosin *in vitro* motility under load. Arch Biochem Biophys. (2015) 580:14–21. 10.1016/j.abb.2015.06.014 26116789 PMC4790447

[27] Greenberg MJ Kazmierczak K Szczesna-Cordary D Moore JR. Cardiomyopathy-linked myosin regulatory light chain mutations disrupt myosin strain-dependent biochemistry. Proc Natl Acad Sci USA. (2010) 107:17403–8. 10.1073/pnas.1009619107 20855589 PMC2951453

[28] Schmid M Toepfer CN. Cardiac myosin super relaxation (SRX): a perspective on fundamental biology, human disease and therapeutics. Biol Open. (2021) 10:bio057646. 10.1242/bio.057646 33589442 PMC7904003

[29] Wang Y Xu Y Kerrick WGL Wang Y Guzman G Diaz-Perez Z. Prolonged Ca^2+^ and force transients in myosin RLC transgenic mouse fibers expressing malignant and benign FHC mutations. J Mol Biol. (2006) 361:286–99. 10.1016/j.jmb.2006.06.018 16837010

[30] Yuan CC Kazmierczak K Liang J Zhou Z Yadav S Gomes AV. Sarcomeric perturbations of myosin motors lead to dilated cardiomyopathy in genetically modified MYL2 mice. Proc Natl Acad Sci USA. (2018) 115:E2338–47. 10.1073/pnas.1716925115 29463717 PMC5877945

[31] Szczesna-Cordary D Jones M Moore JR Watt J Kerrick WGL Xu Y. Myosin regulatory light chain E22K mutation results in decreased cardiac intracellular calcium and force transients. FASEB J. (2007) 21:3974–85. 10.1096/fj.07-8630com 17606808

[32] Fiske CH Subbarow Y. The colorimetric determination of phosphorus. J Biol Chem. (1925) 66:375–400. 10.1016/S0021-9258(18)84756-1

[33] Kazmierczak K Xu Y Jones M Guzman G Hernandez OM Kerrick WGL. The role of the N-terminus of the myosin essential light chain in cardiac muscle contraction. J Mol Biol. (2009) 387:706–25. 10.1016/j.jmb.2009.02.006 19361417 PMC3068778

[34] Pant K Watt J Greenberg M Jones M Szczesna-Cordary D Moore JR. Removal of the cardiac myosin regulatory light chain increases isometric force production. FASEB J. (2009) 23:3571–80. 10.1096/fj.08-126672 19470801 PMC2747675

[35] Yuan CC Kazmierczak K Liang J Ma W Irving TC Szczesna-Cordary D. Molecular basis of force-pCa relation in MYL2 cardiomyopathy mice: role of the super-relaxed state of myosin. Proc Natl Acad Sci USA. (2022) 119:e2110328119. 10.1073/pnas.2110328119 35177471 PMC8872785

[36] Hooijman P Stewart MA Cooke R. A new state of cardiac myosin with very slow ATP turnover: a potential cardioprotective mechanism in the heart. Biophys J. (2011) 100:1969–76. 10.1016/j.bpj.2011.02.061 21504733 PMC3077696

[37] Yang J Yan R Roy A Xu D Poisson J Zhang Y. The I-TASSER suite: protein structure and function prediction. Nat Methods. (2015) 12:7–8. 10.1038/nmeth.3213 25549265 PMC4428668

[38] Stewart MA Franks-Skiba K Chen S Cooke R. Myosin ATP turnover rate is a mechanism involved in thermogenesis in resting skeletal muscle fibers. Proc Natl Acad Sci USA. (2010) 107:430–5. 10.1073/pnas.0909468107 19966283 PMC2806748

[39] Alamo L Qi D Wriggers W Pinto A Zhu J Bilbao A. Conserved intramolecular interactions maintain myosin interacting-heads motifs explaining tarantula muscle super-relaxed state structural basis. J Mol Biol. (2016) 428:1142–64. 10.1016/j.jmb.2016.01.027 26851071 PMC4826325

[40] Garfinkel AC Seidman JG Seidman CE. Genetic pathogenesis of hypertrophic and dilated cardiomyopathy. Heart Fail Clin. (2018) 14:139–46. 10.1016/j.hfc.2017.12.004 29525643 PMC5851453

[41] Sitbon YH Kazmierczak K Liang J Yadav S Veerasammy M Kanashiro-Takeuchi RM. Ablation of the N terminus of cardiac essential light chain promotes the super-relaxed state of myosin and counteracts hypercontractility in hypertrophic cardiomyopathy mutant mice. FEBS J. (2020) 287:3989–4004. 10.1111/febs.15243 32034976 PMC7888128

[42] Yadav S Szczesna-Cordary D. Pseudophosphorylation of cardiac myosin regulatory light chain: a promising new tool for treatment of cardiomyopathy. Biophys Rev. (2017) 9:57–64. 10.1007/s12551-017-0248-8 28510043 PMC5418495

[43] van der Velden J Papp Z Boontje NM Zaremba R de Jong JW Janssen PML. The effect of myosin light chain 2 dephosphorylation on Ca^2+^-sensitivity of force is enhanced in failing human hearts. Cardiovasc Res. (2003) 57:505–14. 10.1016/S0008-6363(02)00662-4 12566123

[44] van der Velden J Papp Z Zaremba R Boontje NM de Jong JW Owen VJ. Increased Ca^2+^-sensitivity of the contractile apparatus in end-stage human heart failure results from altered phosphorylation of contractile proteins. Cardiovasc Res. (2003) 57:37–47. 10.1016/S0008-6363(02)00606-5 12504812

[45] van der Velden J Papp Z Boontje NM Zaremba R de Jong JW Janssen PM. Myosin light chain composition in non-failing donor and end-stage failing human ventricular myocardium. Adv Exp Med Biol. (2003) 538:3–15. 10.1007/978-1-4419-9029-7_1 15098650

[46] Scruggs SB Hinken AC Thawornkaiwong A Robbins J Walker LA de Tombe PP. Ablation of ventricular myosin regulatory light chain phosphorylation in mice causes cardiac dysfunction in situ and affects neighboring myofilament protein phosphorylation. J Biol Chem. (2009) 284:5097–106. 10.1074/jbc.M807414200 19106098 PMC2643522

[47] Sheikh F Ouyang K Campbell SG Lyon RC Chuang J Fitzsimons D. Mouse and computational models link Mlc2v dephosphorylation to altered myosin kinetics in early cardiac disease. J Clin Invest. (2012) 122:1209–21. 10.1172/JCI61134 22426213 PMC3314469

[48] Muthu P Kazmierczak K Jones M Szczesna-Cordary D. The effect of myosin RLC phosphorylation in normal and cardiomyopathic mouse hearts. J Cell Mol Med. (2012) 16:911–9. 10.1111/j.1582-4934.2011.01371.x 21696541 PMC3193868

[49] Abraham TP Jones M Kazmierczak K Liang H-Y Pinheiro AC Wagg CS. Diastolic dysfunction in familial hypertrophic cardiomyopathy transgenic model mice. Cardiovasc Res. (2009) 82:84–92. 10.1093/cvr/cvp016 19150977 PMC2721639

[50] Kerrick WGL Kazmierczak K Xu Y Wang Y Szczesna-Cordary D. Malignant familial hypertrophic cardiomyopathy D166V mutation in the ventricular myosin regulatory light chain causes profound effects in skinned and intact papillary muscle fibers from transgenic mice. FASEB J. (2009) 23:855–65. 10.1096/fj.08-118182 18987303 PMC2653985

[51] Sitbon YH Diaz F Kazmierczak K Liang J Wangpaichitr M Szczesna-Cordary D. Cardiomyopathic mutations in essential light chain reveal mechanisms regulating the super relaxed state of myosin. J Gen Physiol. (2021) 153:e202012801. 10.1085/jgp.202012801 34014247 PMC8142263

[52] Spudich JA. Three perspectives on the molecular basis of hypercontractility caused by hypertrophic cardiomyopathy mutations. Pflugers Arch. (2019) 471:701–17. 10.1007/s00424-019-02259-2 30767072 PMC6475635

